# Characteristics of subclinical tuberculosis compared to active symptomatic tuberculosis using nationwide registry cohort in Korea: prospective cohort study

**DOI:** 10.3389/fpubh.2023.1275125

**Published:** 2023-12-05

**Authors:** Yun-Jeong Jeong, Jae Seuk Park, Hyung Woo Kim, Jinsoo Min, Yousang Ko, Jee Youn Oh, Eun Hye Lee, Bumhee Yang, Joong Hyun Ahn, Jin Woo Kim, Yong Il Hwang, Kwang Joo Park, Sung Soon Lee, Ju Sang Kim, Hyeon-Kyoung Koo

**Affiliations:** ^1^Division of Pulmonary and Critical Care Medicine, Department of Internal Medicine, Dongguk University Ilsan Hospital, Goyang, Republic of Korea; ^2^Division of Pulmonary Medicine, Department of Internal Medicine, Dankook University College of Medicine, Cheonan, Republic of Korea; ^3^Division of Pulmonary and Critical Care Medicine, Department of Internal Medicine, Incheon St. Mary's Hospital, College of Medicine, The Catholic University of Korea, Seoul, Republic of Korea; ^4^Division of Pulmonary and Critical Care Medicine, Department of Internal Medicine, Seoul St. Mary's Hospital, College of Medicine, The Catholic University of Korea, Seoul, Republic of Korea; ^5^Division of Pulmonary, Allergy and Critical Care Medicine, Department of Internal Medicine, Kangdong Sacred Heart Hospital, Hallym University College of Medicine, Seoul, Republic of Korea; ^6^Division of Pulmonary, Allergy and Critical Care Medicine, Department of Internal Medicine, Korea University Guro Hospital, Korea University College of Medicine, Seoul, Republic of Korea; ^7^Division of Pulmonology, Allergy and Critical Care Medicine, Department of Internal Medicine, Yongin Severance Hospital, Yonsei University College of Medicine, Seoul, Republic of Korea; ^8^Division of Pulmonary and Critical Care Medicine, Department of Internal Medicine, Chungbuk National University Hospital, Chungbuk National University College of Medicine, Cheongju, Republic of Korea; ^9^Division of Pulmonary and Critical Care Medicine, Department of Internal Medicine, Uijeongbu St. Mary's Hospital, College of Medicine, The Catholic University of Korea, Uijeongbu, Republic of Korea; ^10^Department of Pulmonary, Allergy and Critical Care Medicine, Hallym University Sacred Heart Hospital, Anyang, Republic of Korea; ^11^Department of Pulmonary and Critical Care Medicine, Ajou University School of Medicine, Suwon, Republic of Korea; ^12^Division of Pulmonary and Critical Care Medicine, Department of Internal Medicine, Ilsan Paik Hospital, Inje University College of Medicine, Goyang, Republic of Korea

**Keywords:** tuberculosis, subclinical, active tuberculosis, symptom, comorbidities

## Abstract

**Objective:**

The clinical manifestations of tuberculosis (TB) range from asymptomatic to disseminated depending on the microbiological and immunological status, making the diagnosis challenging. To improve our understanding of the disease progression mechanism, we aimed to identify the characteristics of subclinical TB and important predictors of symptom development.

**Methods:**

From July 2018 to June 2019, we systemically collected data from the National Surveillance System of South Korea on patients with pulmonary TB, and compared the characteristics of subclinical and active symptomatic TB patients.

**Results:**

A total of 4,636 patients with pulmonary TB were included, and the prevalence of subclinical TB was 37.1% (1,720/4,636). In subclinical TB patients, the positivity rates of acid-fast bacilli (AFB) smear and culture were 16.2 and 50.2%, respectively. Subclinical TB patients were younger (55.6 ± 19.2 vs. 60.7 ± 19.5, *P* < 0.001), had a higher body mass index (21.7 ± 3.1 vs. 21.0 ± 3.5, *P* < 0.001), less under Medicaid support, and had lower rates of chronic lung disease, AFB smear and culture positivity, and bilateral disease. Regarding the characteristic differences of individual TB-related symptoms, age was positively associated with dyspnoea and general weakness but negatively associated with chest pain, haemoptysis, and weight loss. Male patients were more prone to weight loss. Chronic lung disease was related to symptoms including cough/phlegm, dyspnoea, and haemoptysis, while autoimmune diseases were associated with fever and weight loss.

**Conclusions:**

The development of TB-related symptoms was associated with microbiological burden and clinical characteristics including underlying comorbidities, which should be evaluated carefully.

## Introduction

Tuberculosis (TB) is an unresolved public health problem, a major cause of ill health, and one of the leading causes of death worldwide (1). Unfortunately, TB exhibits varied clinical manifestations, making its diagnosis challenging. Occasionally, some patients do not exhibit significant symptoms until they have a substantial disease burden (2). A recent study found that 30% of TB patients diagnosed in the community-basis were completely asymptomatic (3). Those patients at risk of delayed diagnosis are also at risk of being a potential source of infection (4). In South Korea, screening for TB using chest radiography is regularly performed for health insurance subscribers as part of their health examination (5). In addition, new employees of healthcare institutions, schools, and social welfare facilities are required to undergo TB screening tests (6). The specific infectivity of subclinical TB is unclear; however, it is contagious and may play a significant role in TB transmission (7).

This study aimed to investigate the prevalence of subclinical TB and compare the clinical characteristics and microbiological burden in subclinical and active symptomatic TB patients to better understand the population in the gray zone. Additionally, to enhance comprehension of the disease progression process from asymptomatic to overt symptomatic disease, we attempted to describe the characteristic differences regarding specific TB-related symptoms.

## Materials and methods

### Study participants

In South Korea, all physicians are required to report the diagnosis and treatment of TB at the time of the initial diagnosis, and TB nurse specialists monitor until treatment completion as part of the national public-private mix (PPM) TB control project. We constructed the “Korea TB cohort database,” a prospective observational registry database of the reported TB patients (8). Between July 2018 and June 2019, data on patients diagnosed with pulmonary TB were collected from all PPM hospitals. Baseline characteristics were recorded such as age, sex, body mass index (BMI), smoking and alcohol consumption history, history of TB, comorbidities, and TB-related symptoms including cough/phlegm, dyspnoea, chest pain, haemoptysis, fever, general weakness, and weight loss. In addition, microbiological test findings, including sputum acid-fast bacilli (AFB) smear and culture, were collected. AFB culture positivity referred to cases with cultured *Mycobacterium tuberculosis* from respiratory specimens; *non-tuberculous mycobacteria* were excluded. The radiographic findings of presence or absence of cavities and severity of disease were determined using chest radiography or computed tomography.

### Definitions

Subclinical TB was defined as disease caused by *Mycobacterium tuberculosis* without any TB-related symptoms but with detectable abnormalities in radiologic or microbiological assays that could be used to diagnose active TB disease (2). Active symptomatic TB was defined as TB with at least one of the following TB-related symptoms: including cough/phlegm, dyspnoea, chest pain, haemoptysis, fever, general weakness, or weight loss with radiological or microbiological evidence of *M. tuberculosis* (9). The presence of symptoms was evaluated by TB nurse specialists and attending physicians. In South Korea, chest radiography is regularly performed for the general population as part of health checkups or pre-employment examinations. If any radiologic abnormalities are detected, additional evaluation including sputum microbiological testing is conducted at the local public health center or referral hospitals. During this process, active TB could be diagnosed even without typical TB-related symptoms, and such cases were categorized as subclinical TB. The subsequent diagnostic pathway after suspicion of TB was not different in subclinical and symptomatic TB groups.

### Ethical considerations

This study was conducted in accordance with the principles of the Declaration of Helsinki. The Institutional Review Board of Ilsan Paik Hospital, Inje University approved the study protocol (IRB No. ISPAIK 2021-08-012) and waived the need for informed consent since none of the patients was at risk. The Korea Disease Control and Prevention Agency (KDCA) is authorized to collect and analyse surveillance data for public health and research purposes. The KDCA approved data use and provided them without personal identification information.

### Statistical analyses

Patient characteristics were presented as mean and standard deviation for continuous variables and relative frequencies for categorical variables. Continuous variables were compared using *t-*tests or analysis of variance, and categorical variables were compared using the chi-square or Fisher's exact test. A correlation network was constructed using Pearson's correlation, wherein each variable was represented by a specific node, and the node size indicated the prevalence. Links between nodes indicated significant associations (*P* < 0.05), and the edge thickness represented the strength of correlation (Pearson's R coefficient), with blue and pink indicating positive and negative correlations, respectively. The igraph package was used to visualize the correlation networks. The change in TB-related symptoms with age was fitted by the locally weighted scatterplot smoothing method, a non-parametric strategy for fitting a smooth curve through points in a scatter plot. Multivariable analysis for symptom development was performed using logistic regression. Each model for TB-related symptoms incorporated independent variables such as age, sex, BMI, and significant variables in each univariable analysis at *P* values <0.1. The best model was selected using the backward elimination method. All statistical analyses were performed using R software (version 4.3.1, http://www.r-project.org/).

## Results

### Baseline characteristics

**Figure 1:**
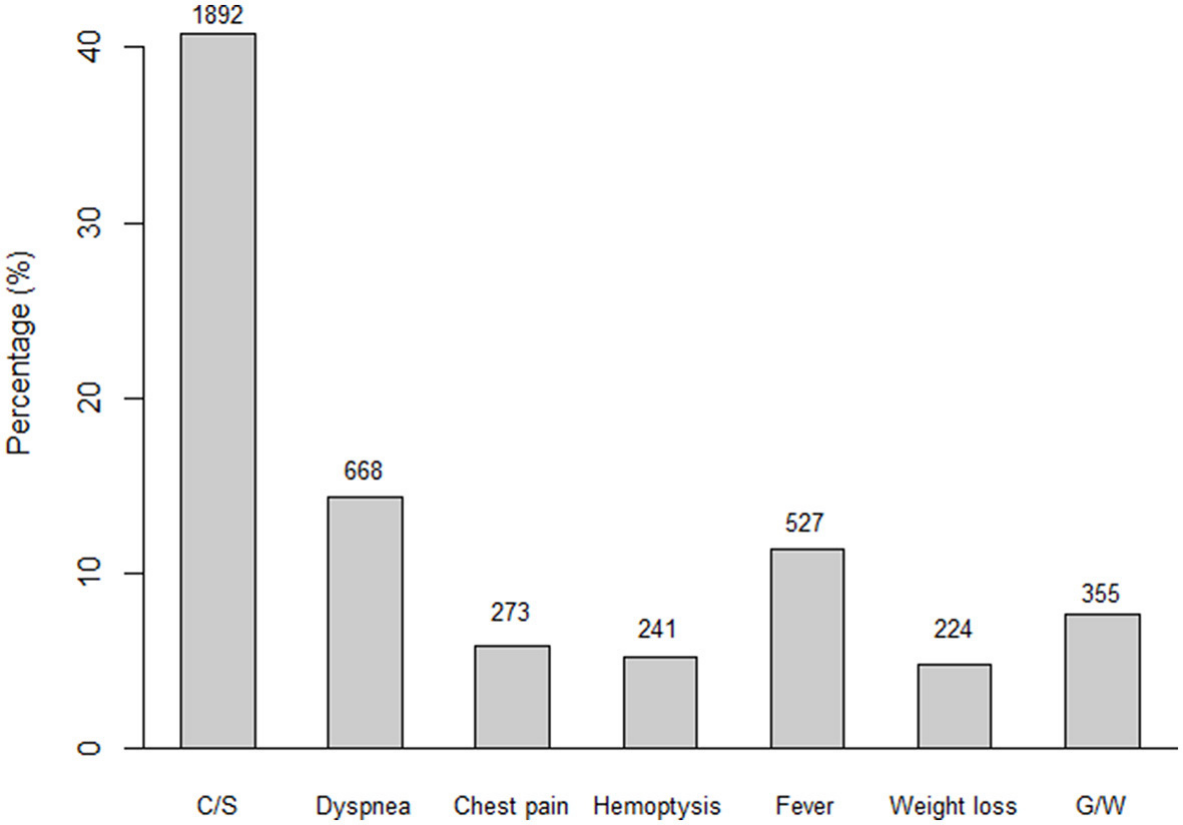
Frequencies of symptoms related to tuberculosis. A total of 4,636 patients were diagnosed with pulmonary TB and 1,720 (37.1%) patients were asymptomatic subclinical TB status. Among the active TB patients, 1,892 (40.8%), 668 (14.4%), 273 (5.9%), 241 (5.2%), 527 (11.4%), 224 (4.8%), and 355 (7.7%) had cough/phlegm, dyspnoea, chest pain, haemoptysis, fever, general weakness, and weight loss, respectively. C/S, cough/sputum; G/W, general weakness; Wt, weight.

During the study period, 4,636 patients with pulmonary TB were enrolled. The mean age was 58.8 years, and 2,898 (62.5%) were men. There were 3,748 (80.8%) newly diagnosed cases, 737 (15.9%) recurrent cases, 10 (0.2%) re-treated cases after failure, and 74 (1.6%) re-treated cases after treatment cessation. There were 1,182 (29.3%) AFB smear-positive, 1,726 (49.4%) TB-polymerase chain reaction (PCR)-positive, and 2,422 (60.9%) AFB culture-positive patient samples. Table 1 summarizes the demographic and clinical characteristics of the participants. Overall, 1,720 (37.1%) patients were asymptomatic, while 2,916 (62.9 %) had one or more symptoms. Among TB-related symptoms, cough/phlegm was observed in 1,892 (40.8%) participants, dyspnoea in 688 (14.8%), chest pain in 273 (5.9%), haemoptysis in 241 (5.2%), fever in 527 (11.4%), general weakness in 224 (4.8%), and weight loss in 355 (7.7%) (Figure 1). A total of 1,071 (23.1%) patients had more than two symptoms. Venn diagram for each symptom is drawn (Supplementary Figure S1). Subclinical TB patients were younger (55.6 ± 19.2 vs. 60.7 ± 19.5, *P* < 0.001), had a higher BMI (21.7 ± 3.1 vs. 21.0 ± 3.5, *P* < 0.001), consumed less alcohol, and were less under Medicaid support. Comorbidities, including diabetes and chronic lung and brain disease, were less frequent in patients with subclinical TB. Radiographic findings revealed that active TB group had more cavitary lesions (15.3 vs. 21.5%, *P* < 0.001) and bilateral involvement (23.6 vs. 37.9%, *P* < 0.001). Positivity rates of AFB smear, AFB culture, and TB-PCR in subclinical TB patients were 16.2, 50.2, and 33.8%, respectively, which were lower than those in active TB patients. The frequency of isoniazid or rifampin resistance was not different. In multivariable analysis, younger age (odds ratio [OR] = 0.99, 95% confidence interval [CI] = 0.98–0.99), higher BMI (OR = 1.04, 95% CI = 1.02–1.06), medical insurance subscriber (OR = 1.35, 95% CI = 1.05–1.72), absence of chronic lung disease (OR = 0.56, 95% CI = 0.39–0.82), negative AFB smear (OR = 2.35, 95% CI = 1.95–2.83), negative AFB culture (OR = 1.50, 95% CI = 1.29–1.75), and unilateral disease (OR = 1.46, 95% CI = 1.24–1.72) were significantly associated with subclinical TB.

**Table 1:** Baseline characteristics of enrolled patients with pulmonary tuberculosis.

		**Total (*N =* 4,636)**	**Subclinical TB (*N =* 1,720)**	**Active TB (*N =* 2,916)**	***P*-value**
Age (years)		58.8 ± 19.8	55.6 ± 19.2	60.7 ± 19.5	< 0.001
Male sex (*n*, %)		2,898 (62.5%)	1,091 (63.4%)	1,807 (62.0%)	0.336
Body mass index, kg/m^2^		21.3 ± 3.4	21.7 ± 3.1	21.0 ± 3.5	< 0.001
Medicaid support (*n*, %)		462 (10.6%)	140 (8.7%)	322 (11.8%)	0.002
Smoking	Never (*n*, %)	2,706 (58.4%)	997 (58.0%)	1,709 (58.6%)	0.564
	Ex- (*n*, %)	914 (19.7%)	332 (19.3%)	582 (20.0%)	
	Current (*n*, %)	1,016 (21.9%)	391 (22.7%)	625 (21.4%)	
Alcohol habbit	None (*n*, %)	2,470 (60.3%)	849 (57.2%)	1,621 (62.1%)	< 0.001
	Social (*n*, %)	1313 (32.1%)	535 (36.0%)	778 (29.8%)	
	Heavy (*n*, %)	312 (7.6%)	101 (6.8%)	221 (8.1%)	
Comorbidities	Diabetes (*n*, %)	956 (20.6%)	327 (19.0%)	629 (21.6%)	0.041
	Chronic lung disease (*n*, %)	238 (5.1%)	52 (3.0%)	186 (6.4%)	< 0.001
	Chronic heart disease (*n*, %)	221 (4.8%)	68 (4.0%)	153 (5.2%)	0.054
	Chronic liver disease (*n*, %)	96 (2.1%)	49 (2.8%)	47 (1.6%)	0.006
	Chronic kidney disease (*n*, %)	130 (2.8%)	50 (2.9%)	80 (2.7%)	0.815
	Chronic brain disease (*n*, %)	396 (8.5%)	122 (7.1%)	274 (9.4%)	0.008
	Malignancy (*n*, %)	456 (9.8%)	215 (12.5%)	241 (8.3%)	< 0.001
	Autoimmune disease (*n*, %)	53 (1.1%)	19 (1.1%)	34 (1.1%)	0.963
	Long-term steroid use (*n*, %)	17 (0.4%)	3 (0.2%)	14 (0.5%)	0.158
	TNF blocker use (*n*, %)	8 (0.2%)	3 (0.2%)	5 (0.2%)	>0.999
	Gastrectomy (*n*, %)	50 (1.1%)	20 (1.2%)	30 (1.0%)	0.780
Radiographic characteristics	Cavitary disease (*n*, %)	869 (19.2%)	254 (15.3%)	615 (21.5%)	< 0.001
	Bilateral disease (*n*, %)	1,435 (32.7%)	376 (23.6%)	1,059 (37.9%)	< 0.001
Microbiologic characteristics	AFB smear (+) (*n*, %)	1,182 (29.3%)	219 (16.2%)	963 (35.9%)	< 0.001
	TB-PCR (+) (*n*, %)	1,726 (49.4%)	383 (33.8%)	1,343 (56.9%)	< 0.001
	AFB culture (+) (*n*, %)	2,422 (60.9%)	668 (50.2%)	1,754 (66.3%)	< 0.001
	INH resistance (*n*, %)	283 (6.1%)	101 (5.9%)	182 (6.2%)	0.671
	RFP resistance (*n*, %)	99 (2.1%)	40 (2.3%)	59 (2.0%)	0.553

Participants' characteristics were presented as mean and standard deviation for continuous variables and as relative frequencies for categorical variables. Continuous variables were compared using *t-*test, and categorical variables using chi-squared test. TB, tuberculosis; TNF, tumor necrosis factor; ds, disease; AFB, acid-fast bacilli; PCR, polymerase chain reaction.

Demographic and clinical characteristics of each TB-related symptom were compared, as detailed in Supplementary Table S1. Patients with dyspnoea (67.3 ± 17.1 vs. 57.4 ± 19.6, *P* < 0.001), fever (61.0 ± 20.4 vs. 58.5 ± 19.4, *P* = 0.006), or general weakness (69.3 ± 15.8 vs. 58.3 ± 19.6, *P* < 0.001) were older than those with chest pain (52.3 ± 20.6 vs. 59.2 ± 19.4, *P* < 0.001), haemoptysis (53.3 ± 19.8 vs. 59.1 ± 19.5, *P* < 0.001), or weight loss (56.2 ± 19.0 vs. 59.0 ± 19.6, *P* = 0.008). Patients with dyspnoea (20.8 ± 3.8 vs. 21.4 ± 3.3, *P* < 0.001), fever (20.9 ± 3.5 vs. 21.3 ± 3.4, *P* = 0.012), general weakness (19.3 ± 3.4 vs. 21.4 ± 3.3, *P* < 0.001), and weight loss (19.3 ± 3.0 vs. 21.4 ± 3.4, *P* < 0.001) had lower BMIs. Patients experiencing haemoptysis (71.4 vs. 62.0%, *P* = 0.004) and weight loss (73.0 vs. 61.6%, *P* < 0.001) were male predominant. Current smokers were more frequently observed in patients with chest pain (28.6 vs. 21.5%, *P* = 0.019) and haemoptysis (27.8 vs. 21.6%, *P* = 0.044).

Regarding the comorbidities, chronic lung (12.6 vs. 3.9%, *P* < 0.001), heart (8.7 vs. 4.1%, *P* < 0.001), and brain (12.1 vs. 7.9%, *P* < 0.001) diseases, and a history of gastrectomy (1.9 vs. 0.9%, *P* = 0.032) were more common in the group with dyspnoea. Diabetes (13.2 vs. 21.1%, *P* = 0.002) and chronic brain disease (4.0 vs. 8.8%, *P* = 0.008) were less common in patients with chest pain, whereas chronic lung disease (8.7 vs. 4.9%, *P* = 0.015) was more common in those with haemoptysis. Chronic brain (14.4 vs. 7.8%, *P* < 0.001) and autoimmune diseases (2.5 vs. 1.0%, *P* = 0.005) were more prevalent in patients with fever. In contrast, patients with general weakness had more comorbidities, including diabetes (25.9 vs. 20.4%, *P* = 0.056), chronic kidney (6.2 vs. 2.6%, *P* = 0.003) and brain diseases (16.1 vs. 8.2%, *P* < 0.001), malignancies (13.8 vs. 9.6%, *P* = 0.051), and long-term steroid use (1.8 vs. 0.3%, *P* = 0.002). Patients with weight loss had fewer incidences of chronic heart (1.7 vs. 5.0%, *P* = 0.007), kidney (0.8 vs. 3.0%, *P* = 0.031), brain diseases (5.6 vs. 8.8%, *P* = 0.052), and malignancies (5.6 vs. 10.25, *P* = 0.007).

**Figure 2:**
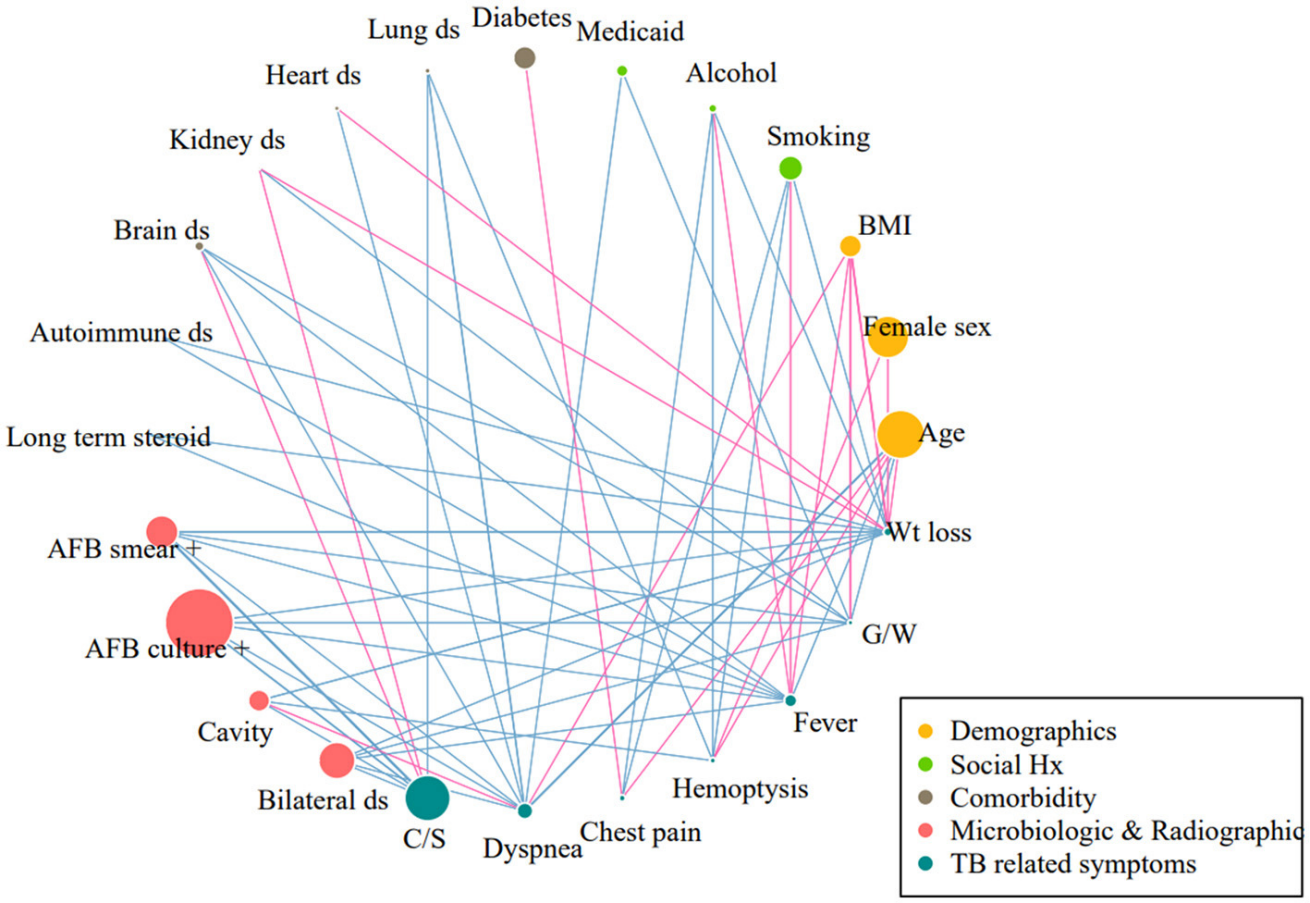
Correlation network between each symptom and demographic, microbiologic, and radiographic features. Correlation matrix was constructed using the Pearson correlation. Each variable was represented as a specific node: the diameter of node was proportional to the prevalence of the variables. The links between nodes indicate a statistically significant association (*P* < 0.05). The thickness of the edges correlated with the strength of their association. Blue indicates positive correlation and pink indicates negative correlation. Only correlations existing with TB-related symptoms are demonstrated. BMI, body mass index; AFB, acid-fast bacilli; C/S, cough/sputum; G/W, general weakness; Wt, weight.

Regarding radiographic features, cavitary lesions were more frequent in patients with cough/phlegm (24.0 vs. 15.9%, *P* < 0.001), haemoptysis (36.1 vs. 18.3%, *P* < 0.001), and weight loss (34.8 vs. 17.9%, *P* < 0.001), but less frequent in those with dyspnoea (16.4 vs. 19.7%, *P* = 0.050). Bilateral lung involvement was more frequent in patients with dyspnoea (43.4 vs. 30.9%, *P* < 0.001), fever (41.6 vs. 31.6%, *P* < 0.001), general weakness (52.1 vs. 31.7%, *P* < 0.001), and weight loss (49.0 vs. 31.3%, *P* < 0.001). Correlation matrix and network demonstrating the inter-relationship between clinical features and each symptom are shown in Supplementary Figure S2 and Figure 2, respectively.

### Factors associated with symptoms development

**Figure 3:**
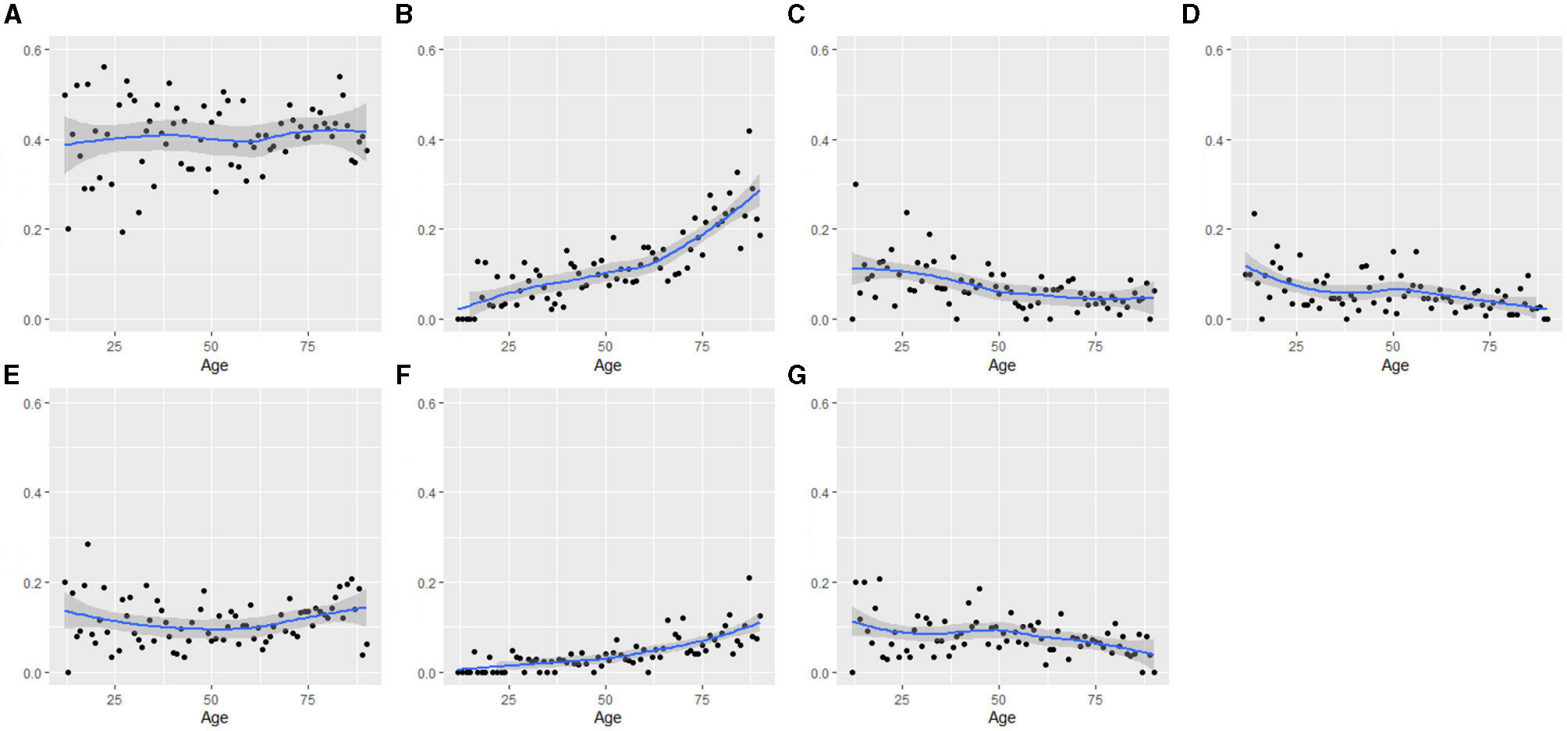
Changes of proportion of symptoms according to age: **(A)** cough/phlegm, **(B)** dyspnoea, **(C)** chest pain, **(D)** haemoptysis, **(E)** fever, **(F)** general weakness, and **(G)** weight loss. Change of proportion of TB-related symptom according to age was fitted by LOWESS method. The value of x- axis is age, and y- axis is proportion of TB-related symptoms. OR for cough/phlegm = 1.002 (*P* = 0.358); OR for dyspnoea = 1.029 (*P* < 0.001); OR for chest pain = 0.980 (*P* < 0.001); OR for haemoptysis = 0.982 (*P* < 0.001); OR for fever = 1.004 (*P* = 0.177); OR for general weakness = 1.032 (*P* < 0.001); and OR for weight loss = 0.991 (*P* = 0.003).

Univariable logistic regression analysis was performed for each symptom (Supplementary Table S2). Since age showed distinct effects on different symptoms, changes in the proportion of each symptom according to age were drawn (Figure 3). The proportion of dyspnoea, fever, and general weakness increased with age, but that of chest pain, haemoptysis, and weight loss decreased. Cough/phlegm, on the other hand, did not show a significant correlation with age.

In multivariable analysis for the asymptomatic subclinical disease, younger age (OR = 0.99, 95% CI = 0.98–1.00), higher BMI (OR = 1.04, 95% CI = 1.10–1.06), under Medicare support (OR = 1.35, 95% CI = 1.05–1.72), absence of chronic lung disease (OR = 1.78, 95% CI = 1.23–2.58), negative sputum AFB smear (OR = 2.35, 95% CI = 1.95–2.83), and culture (OR = 1.50, 95% CI = 1.29–1.75) were significantly associated. Table 2 summarizes the multivariate analysis results for each symptom. Cough/phlegm were independently associated with female sex (OR = 1.28, 95% CI = 1.11–1.47), higher BMI (OR = 1.02, 95% CI = 1.00–1.05), underlying chronic lung disease (OR = 1.39, 95% CI = 1.04–1.86), positive sputum AFB smear (OR = 2.14, 95% CI = 1.84–2.50) and culture (OR = 1.53, 95% CI = 1.33–1.77), cavitary lesions (OR = 1.39, 95% CI = 1.17–1.64), and bilateral disease (OR = 1.17, 95% CI = 1.01–1.35). For dyspnoea, advanced age (OR = 1.03, 95% CI = 1.02–1.03), Medicaid support (OR = 1.45, 95% CI = 1.12–1.88), underlying chronic lung disease (OR = 2.90, 95% CI = 2.14–3.93), chronic heart disease (OR = 1.46, 95% CI = 1.03–2.07), and bilateral disease (OR = 1.57, 95% CI = 1.30–1.88) were selected. For chest pain, younger age (OR = 0.98, 95% CI = 0.98–0.99), absence of diabetes (OR = 0.64, 95% CI = 0.43–0.95), and sputum AFB smear positivity (OR = 1.36, 95% CI = 1.03–1.79) were chosen. For haemoptysis, younger age (OR = 0.99, 95% CI = 0.98–0.99), heavy alcohol consumption (OR = 2.18, 95% CI = 1.49–3.21), chronic lung disease (OR = 2.18, 95% CI = 1.31–3.63), and cavitary lesions (OR = 2.20, 95% CI = 1.63–2.96) were significant. For fever, non-current smokers (OR = 0.63, 95% CI = 0.48–0.83), Medicaid support (OR = 1.40, 95% CI = 1.04–1.87), chronic brain disease (OR = 1.83, 95% CI = 1.36–2.46), autoimmune disease (OR = 2.33, 95% CI = 1.12–4.83), sputum AFB smear positivity (OR = 1.28, 95% CI = 1.04–1.59), and bilateral disease (OR = 1.36, 95% CI = 1.10–1.67) were chosen. For general weakness, older age (OR = 1.03, 95% CI = 1.02–1.04), lower BMI (OR = 0.86, 95% CI = 0.82–0.90), heavy alcohol consumption (OR = 2.26, 95% CI = 1.42–3.58), Medicaid support (OR = 2.00, 95% CI = 1.35–2.97), long term steroid use (OR = 5.09, 95% CI = 1.06–24.47), and sputum AFB positivity (OR = 1.96, 95% CI = 1.44–2.68) were included. For weight loss, younger age (OR = 0.99, 95% CI = 0.98–0.99), male sex (OR = 1.66, 95% CI = 1.26–2.18), lower BMI (OR = 0.83, 95% CI = 0.80–0.87), absence of chronic heart disease (OR = 0.30, 95% CI = 0.12–0.77), presence of autoimmune disease (OR = 3.15, 95% CI = 1.39–7.13), positivity rates of AFB smear (OR = 1.78, 95% CI = 1.37–2.32) and culture (OR = 1.38, 95% CI = 1.04–1.82), cavitary lesions (OR = 1.50, 95% CI = 1.15–1.96), and bilateral disease (OR = 1.48, 95% CI = 1.15–1.90) were selected. Positive AFB smear results were independently associated with symptoms of cough/phlegm (OR = 2.51, 95% CI = 2.17–2.89), dyspnoea (OR = 1.34, 95% CI = 1.17–1.61), haemoptysis (OR = 1.32, 95% CI = 0.98–1.78), general weakness (OR = 2.52, 95% CI = 1.88–3.89), and weight loss (OR = 2.10, 95% CI = 1.66–2.66). Positive AFB culture results were also associated with symptoms of cough/phlegm (OR = 1.85, 95% CI = 1.62–2.11), dyspnoea (OR = 1.20, 95% CI = 1.00–1.44), general weakness (OR = 1.85, 95% CI = 1.34–2.55), and weight loss (OR = 1.59, 95% CI = 1.22–2.06). The counted number of TB-related symptoms was classified as follows: 0, 1, 2, 3, and ≥4. The positivity of the AFB smear (OR = 1.60, 95% CI = 1.49–1.72) and culture (OR = 1.39, 95% CI = 1.30–1.49) significantly increased with the number of TB-related symptoms (Supplementary Figure S3).

**Table 2:** Multivariable analysis for TB-related symptoms: (A) respiratory and (B) systemic symptoms.

**(A)**	**Adjusted OR**	**95% CI**
**Cough/phlegm**		
Female sex	1.280	1.113–1.473
Body mass index	1.024	1.004–1.045
Chronic lung disease	1.391	1.041–1.858
AFB smear positivity	2.144	1.837–2.501
AFB culture positivity	1.530	1.326–1.765
Cavitary disease	1.388	1.172–1.644
Bilateral disease	1.168	1.011–1.349
**Dyspnoea**		
Age	1.028	1.022–1.034
Medicaid support	1.449	1.115–1.882
Chronic lung disease	2.900	2.139–3.932
Chronic heart disease	1.464	1.034–2.072
Bilateral disease	1.565	1.303–1.878
**Chest pain**		
Age	0.982	0.976–0.988
Diabetes	0.636	0.427–0.947
AFB culture positivity	1.356	1.026–1.793
**Haemoptysis**		
Age	0.985	0.978–0.992
Heavy alcoholics	2.184	1.487–3.209
Chronic lung disease	2.176	1.305–3.627
Cavitary disease	2.197	1.633–2.957
**(B)**	**Adjusted OR**	**95% CI**
**Fever**		
Current smoking	0.632	0.482–0.829
Medicaid support	1.395	1.042–1.867
Chronic brain disease	1.832	1.364–2.461
Autoimmune disease	2.325	1.118–4.832
AFB smear positivity	1.283	1.035–1.592
Bilateral disease	1.357	1.102–1.671
**General weakness**		
Age	1.032	1.021–1.042
Body mass index	0.858	0.817–0.900
Heavy alcoholics	2.255	1.423–3.575
Medicaid support	2.004	1.352–2.972
Long term steroid use	5.087	1.057–24.472
AFB smear positivity	1.964	1.440–2.677
**Weight loss**		
Age	0.991	0.985–0.997
Male sex	1.661	1.263–2.179
Body mass index	0.833	0.801–0.867
Chronic heart disease	0.304	0.120–0.774
Autoimmune disease	3.145	1.388–7.125
AFB smear positivity	1.780	1.368–2.316
AFB culture positivity	1.379	1.043–1.824
Cavitary disease	1.498	1.145–1.960
Bilateral disease	1.478	1.152–1.897

The multivariable model was developed using logistic regression including significant variables in univariate analysis at *P* value < 0.1 in addition to age, sex, and BMI. The best model was selected using backward elimination method. AFB, acid-fast bacilli.

## Discussion

Here, we compared the characteristics of subclinical and active symptomatic TB patients and identified factors associated with developing TB-related symptoms. In our cohort, the prevalence of subclinical TB in South Korea was 37.1 %, with positivity rates of AFB smear and culture of 16.2 and 50.2%, respectively. Subclinical TB patients had a lower proportion of AFB smear and culture positivity than active TB patients; however, considerably high rate of AFB positive results were observed, which emphasizes the need to active search for this population that might be potential sources of infection. For each symptom, demographics, underlying comorbidities, radiographic features, and microbiologic burdens were inter-related, suggesting a mechanism for progression to full-blown disease. The most remarkable finding of our study was that each symptom was not only associated with a microbiological burden as represented by sputum AFB smear grade but also with different demographic characteristics, including age, sex, social factors, and various comorbidities.

In a multicentre drug-susceptible TB cohort in Korea, Min et al. (10) reported that the positivity of sputum AFB smear and culture were 13.6 and 46.2%, respectively, similar to our results. Moreover, advanced age was a significant factor for development of active TB. A recent large retrospective cohort study in Canada reported that the proportion of patients aged 15–64 years was higher in subclinical TB (11). The Suzhou study also discovered that the prevalence of subclinical TB decreased substantially with age (12). Likewise, we discovered that subclinical TB patients were significantly younger, however, age was found to act differently for each TB-related symptom. The higher probability of early detection of subclinical TB could be attributable to frequent health check-ups at school or work for younger patients. Considering the distinct effect of age, active surveillance is also required to older population for early TB diagnosis.

Subclinical TB was more prevalent in women in a recent Zambia study (13), whereas Min et al. reported that it was more prevalent in men. We discovered no significant association between subclinical TB and sex; however, the symptoms of cough/phlegm and weight loss were independently associated with the female and male sex, respectively. The association between subclinical TB and sex requires further investigation.

In a study conducted in Zambia (13), the probability of subclinical TB increased with socioeconomic status. Similarly, the proportion of patients with Medicaid support was lower in the subclinical TB group in our study, suggesting a true association between lower socioeconomic status and active symptomatic TB. Haemoptysis and general weakness were more frequently observed in patients who consumed excessive alcohol. In addition, patients receiving Medicaid support experienced more severe symptoms, such as dyspnoea, fever, and general weakness, reflecting a delayed diagnosis due to poor access to medical facilities. This raises the need to pay attention toward this vulnerable population.

The incidence of comorbidities, including diabetes and chronic lung and brain diseases, was lower in the subclinical TB group, suggesting the rapid progression to active TB in patients with impaired immunologic response due to underlying comorbidities. However, definite conclusions cannot be drawn. To the best of our knowledge, no data on the association between comorbidities and TB-related symptoms are available. Diabetes and chronic lung disease are known risk factors for developing and progressing to active TB (14). Conditions with underlying chronic lung disease were significantly associated with respiratory symptoms, such as cough/phlegm, dyspnoea, and haemoptysis. Additionally, autoimmune diseases were more related to systemic symptoms, such as fever and weight loss. Patients with chronic heart disease complained more frequently of dyspnoea but lesser weight loss, probably due to fluid overload. Since symptoms related to TB or the underlying disease might be confusing due to their overlapping nature, a multilateral approach considering comorbidities is required when evaluating TB-related symptoms.

The key strength of our study was the large number of reported TB cases collected systemically across the country, which represented the actual burden of subclinical TB in Korea. Therefore, our study could be a reference for planning government healthcare policies.

Several limitations should be addressed. First, it remains unclear whether the factors associated with active symptomatic TB are related to delayed diagnosis or triggered awareness of the onset of symptoms. Second, our study population was recruited from PPM-affiliated hospitals and excluding patients from non-PPM hospitals could limit generalization. However, approximately 70% of TB patients were treated under the PPM program. Third, we could not analyse laboratory biomarkers because our prospective nationwide cohort registry did not include these variables. Additionally, though sputum AFB culture positivity in subclinical TB patients could reflect infectivity, actual infectivity in this population might be lower due to the absence of cough. Future well-designed prospective studies should be conducted to monitor the clinical, immunological, and transmission outcomes of these patients and their contacts.

In conclusion, the development of TB-related symptoms was associated with microbiological burden and clinical characteristics, including underlying comorbidities, which should be evaluated and monitored carefully.

## Data availability statement

The datasets presented in this article are not readily available because the data that supports the findings of this study are available from the KDCA; however, restrictions apply to the availability of such data, which are not publicly available. Data are however available from the authors upon reasonable request and with permission of the KDCA. Requests to access the datasets should be directed to gusrud9@yahoo.co.kr.

## Ethics statement

This study was conducted in accordance with the principles of the Declaration of Helsinki. The Institutional Review Board of Ilsan Paik Hospital, Inje University approved the study protocol (IRB No. ISPAIK 2021-08-012) and waived the need for informed consent since none of the patients were at risk. The Korea Disease Control and Prevention Agency (KDCA) is authorized to collect and analyze surveillance data for public health and research purposes. The KDCA approved data use and provided them without personal identification information.

## Author contributions

Y-JJ: Investigation, Writing – original draft, Writing – review & editing, Conceptualization. JSP: Conceptualization, Funding acquisition, Investigation, Supervision, Writing – review & editing. HWK: Investigation, Writing – review & editing. JM: Investigation, Conceptualization, Supervision, Validation, Writing – review & editing. YK: Investigation, Writing – review & editing. JYO: Investigation, Writing – review & editing. EHL: Investigation, Writing – review & editing. BY: Investigation, Writing – review & editing. JHA: Investigation, Writing – review & editing. JWK: Investigation, Writing – review & editing. YIH: Investigation, Writing – review & editing. KJP: Investigation, Writing – review & editing. SSL: Investigation, Writing – review & editing. JSK: Funding acquisition, Investigation, Supervision, Writing – review & editing. H-KK: Conceptualization, Formal analysis, Investigation, Writing – original draft, Writing – review & editing, Data curation.
