# Supplementary material for: Characteristics of subclinical tuberculosis compared to active symptomatic tuberculosis using nationwide registry cohort in Korea: prospective cohort study
Source: Front Public Health. 2023 Dec 5;11:1275125. doi: 10.3389/fpubh.2023.1275125 (PMC10732352; doi:10.3389/fpubh.2023.1275125)

**Supplementary data**

Supplementary table S1. Detailed baseline characteristics comparing patients with each symptom

|  |  | **Cough/phlegm** | **Dyspnea** | **Chest pain** | **Hemoptysis** | **Fever** | **General weakness** | **Weight loss** |
| --- | --- | --- | --- | --- | --- | --- | --- | --- |
| Number (%) |  | 1892 (40.8) | 688 (14.8) | 273 (5.9) | 241 (5.2) | 527 (11.4) | 224 (4.8) | 355 (7.7) |
| **Demographics** | |  |  |  |  |  |  |  |
| Age |  | 59.3±19.5 | 67.3±17.1* | 52.4±20.6* | 53.3±19.8* | 61.0±20.4* | 69.3±15.8* | 56.2±19.0* |
| Male sex |  | 1152(60.9%) | 433(64.8%) | 163(59.7%) | 172(71.4%)* | 315(59.8%) | 150(67.0%) | 259(73.0%)* |
| Height |  | 163.0±11.6 | 161.8±9.8* | 163.8±9.8 | 165.9±9.0* | 163.1±15.4 | 162.1±10.2 | 164.7±9.5* |
| Weight |  | 56.6±11.4 | 54.5±11.6* | 57.4±10.8 | 58.6±11.3* | 55.7±11.8* | 50.7±10.2* | 52.5±9.7* |
| BMI |  | 21.3±3.5* | 20.8±3.8* | 21.3±3.3 | 21.2±3.3 | 20.9±3.5* | 19.3±3.4* | 19.3±3.0* |
| **Social factors** | |  |  |  |  |  |  |  |
| Medicaid |  | 190(10.7%) | 87(14.1%)* | 20(8.2%) | 23(10.1%) | 67(13.9%)* | 46(21.7%)* | 44(13.2%) |
| Smoking | Never | 1079(57.0%) | 392(58.7%)* | 142(52.0%)* | 124(51.5%)* | 345(65.5%)* | 130(58.0%) | 172(48.5%)* |
|  | Ex- | 386(20.4%) | 158(23.7%)* | 53(19.4%)* | 50(20.7%)* | 97(18.4%)* | 48(21.4%) | 85(23.9%)* |
|  | Current | 427(22.6%) | 118(17.7%)* | 78(28.6%)* | 67(27.8%)* | 85(16.1%)* | 46(20.5%) | 98(27.6%)* |
| Alcohol | None | 1024(60.4%) | 392(65.4%)* | 131(52.6%)* | 106(48.6%)* | 326(68.2%)* | 128(63.4%)* | 181(55.2%)* |
|  | Social | 532(31.4%) | 157(26.2%)* | 99(39.8%)* | 757(34.4%)* | 117(24.5%)* | 44(21.8%)* | 103(31.4%)* |
|  | Heavy | 140(8.3%) | 50(8.3%)* | 19(7.6%)* | 37(17.0%)* | 35(7.3%)* | 30(14.9%)* | 44(13.4%)* |
| **Comorbidity** | | 1090(57.6%) | 482(72.2%)* | 130(47.6%)* | 122(50.6%)* | 344(65.4%)* | 178(79.5%)* | 197(55.5%) |
|  | Diabetes | 405(21.4%) | 152(22.8%)* | 36(13.2%)* | 55(22.8%) | 109(20.7%) | 58(25.9%) | 85(23.9%) |
|  | Chronic lung ds | 117(6.2%)* | 84(12.6%)* | 12(4.4%) | 21(8.7%)* | 31(5.9%) | 12(5.4%) | 14(3.9%) |
|  | Chronic heart ds | 93(4.9%) | 58(8.7%)* | 14(5.1%) | 8(3.3%) | 32(6.1%) | 13(5.8%) | 6(1.7%)* |
|  | Chronic liver ds | 32(1.7%) | 9(1.3%) | 4(1.5%) | 3(1.2%) | 13(2.5%) | 5(2.2%) | 5(1.4%) |
|  | Chronic kidney ds | 44(2.3%) | 21(3.1%) | 3(1.1%) | 1(0.4%)* | 14(2.7%) | 14(6.2%)* | 3(0.8%)* |
|  | Chronic brain ds | 139(7.3%) | 81(12.1%)* | 11(4.0%)* | 12(5.0%) | 76(14.4%)* | 36(16.1%)* | 20(5.6%) |
|  | Malignant | 137(7.2%)* | 72(10.8%) | 23(8.4%) | 13(5.4%)* | 55(10.4%) | 31(13.8%)* | 20(5.6%)* |
|  | Autoimmune ds | 21(1.1%) | 10(1.5%) | 3(1.1%) | 2(0.8%) | 13(2.5%)* | 5(2.2%) | 8(2.3%) |
|  | Long-term steroid | 6(0.3%) | 4(0.6%) | 2(0.7%) | 0(0%) | 4(0.8%) | 4(1.8%)* | 3(0.8%) |
|  | TNF blocker | 2(0.1%) | 2(0.3%) | 0(0%) | 0(0%) | 2(0.4%) | 0(0%) | 1(0.3%) |
|  | Gastrectomy | 21(1.1%) | 13(1.9%)* | 0(0%) | 1(0.4%) | 4(0.8%) | 3(1.3%) | 3(0.8%) |
|  | TPL | 8(0.4%) | 1(0.1%) | 0(0%) | 0(0%) | 4(0.8%) | 1(0.4%) | 0(0%) |
|  | Others | 740(39.1%) | 324(48.5%)* | 86(31.5%)* | 71(29.5%)* | 231(43.8%)* | 124(55.4%)* | 128(36.1%) |
| **TB type** |  |  |  |  |  |  |  |  |
|  | New case | 1544(81.6%) | 512(76.6%)* | 228(83.5%) | 166(68.9%)* | 434(82.4%) | 179(79.9%) | 287(80.8%) |
|  | Recurred | 295(15.6%) | 124(18.6%)* | 42(15.4%) | 69(28.6%)* | 82(15.6%) | 33(14.7%) | 53(14.9%) |
| **Radiographic features** | |  |  |  |  |  |  |  |
|  | Cavity | 446(24.0%)* | 107(16.4%)* | 52(19.5%) | 86(36.1%)* | 87(16.9%) | 49(22.4%) | 123(34.8%)* |
|  | Bilateral | 680(37.4%) | 278(43.4%)* | 74(28.1%) | 85(37.0%) | 209(41.6%)* | 114(52.1%)* | 171(49.0%)* |
| **Microbiologic features** | |  |  |  |  |  |  |  |
| AFB smear positivity | | 704(40.3%)* | 229(36.5%)* | 67(27.5%) | 81(36.5%)* | 172(34.4%)* | 100(47.6%)* | 166(50.5%)* |
| AFB culture positivity | | 1195(69.2%)* | 409(66.1%) | 157(66.2%) | 121(56.0%) | 326(66.1%)* | 152(73.1%)* | 241(73.7%)* |
| TB PCR positivity | | 926(59.6%)* | 330(58.5%)* | 102(50.2%) | 102(51.8%) | 252(56.1%)* | 119(64.7%)* | 202(68.0%)* |
| INH resistance | | 121(6.4%) | 43(6.4%) | 18(6.6%) | 16(6.6%) | 33(6.3%) | 12(5.4%) | 22(6.2%) |
| RFP resistance | | 37(2.0%) | 14(2.1%) | 8(2.9%) | 6(2.5%) | 9(1.7%) | 7(3.1%) | 7(2.0%) |

Data are presented as number (%) or mean ± standard deviation

* indicates the statistical significance

Abbreviation: BMI, body mass index; ds, disease; TNF, tumor necrosis factor; TPL, transplantation; AFB, acid-fast bacilli; PCR, polymerase chain reaction

Supplementary Table S2. Univariable analysis for development of each symptom

| Univariable | **Cough/phlegm** | | **Dyspnea** | | **Chest pain** | | **Hemoptysis** | | **Fever** |  | **General weakness** | | **Weight loss** | |
| --- | --- | --- | --- | --- | --- | --- | --- | --- | --- | --- | --- | --- | --- | --- |
|  | OR | 95% CI | OR | 95% CI | OR | 95% CI | OR | 95% CI | OR | 95% CI | OR | 95% CI | OR | 95% CI |
| **Age** | 1.002 | 0.998-1.005 | 1.029 | 1.023-1.035 | 0.980 | 0.975-0.986 | 0.982 | 0.975-0.989 | 1.004 | 0.998-1.009 | 1.032 | 1.022-1.041 | 0.991 | 0.985-0.997 |
| **Female sex** | 1.153 | 0.997-1.334 | 0.900 | 0.735-1.120 | 1.002 | 0.775-1.295 | 0.570 | 0.405-0.803 | 1.080 | 0.878-1.328 | 0.922 | 0.668-1.271 | 0.579 | 0.439-0.765 |
| **Body mass index** | 0.999 | 0.979-1.020 | 0.941 | 0.915-0.969 | 1.030 | 0.994-1.067 | 1.007 | 0.963-1.050 | 0.974 | 0.946-1.003 | 0.822 | 0.783-0.863 | 0.819 | 0.786-0.852 |
| **Smoking** | 1.080 | 0.916-1.274 | 0.698 | 0.547-0.891 | 1.462 | 1.113-1.920 | 1.445 | 1.040-2.006 | 0.691 | 0.533-0.897 | 0.897 | 0.620-1.298 | 1.565 | 1.199-2.042 |
| **Heavy alcoholics** | 1.159 | 0.898-1.495 | 1.023 | 0.722-1.449 | 0.952 | 0.599-1.512 | 2.979 | 2.015-4.404 | 1.004 | 0.694-1.452 | 2.177 | 1.414-3.353 | 1.945 | 1.350-2.801 |
| **Medicaid support** | 1.016 | 0.812-1.270 | 1.387 | 1.045-1.841 | 0.555 | 0.340-0.907 | 0.891 | 0.541-1.467 | 1.333 | 0.990-1.796 | 2.391 | 1.638-3.490 | 1.285 | 0.895-1.844 |
| **Diabetes** | 1.109 | 0.935-1.315 | 1.129 | 0.898-1.420 | 0.525 | 0.365-0.757 | 1.125 | 0.789-1.605 | 1.015 | 0.795-1.298 | 1.216 | 0.852-1.734 | 1.188 | 0.891-1.583 |
| **chronic lung ds** | 1.357 | 1.002-1.836 | 3.446 | 2.498-4.754 | 0.554 | 0.281-1.095 | 1.938 | 1.164-3.226 | 0.982 | 0.633-1.525 | 0.908 | 0.456-1.808 | 0.595 | 0.310-1.139 |
| **Chronic heart ds** | 1.041 | 0.752-1.441 | 2.132 | 1.475-3.082 | 0.860 | 0.472-1.567 | 0.412 | 0.151-1.122 | 1.359 | 0.895-2.063 | 0.808 | 0.373-1.753 | 0.262 | 0.096-0.714 |
| **Chronic liver ds** | 0.535 | 0.315-0.909 | 0.639 | 0.290-1.407 | 0.797 | 0.319-1.989 | 0.469 | 0.114-1.924 | 1.274 | 0.683-2.377 | 0.813 | 0.253-2.615 | 0.315 | 0.077-1.291 |
| **Chronic kidney ds** | 0.625 | 0.403-0.969 | 1.332 | 0.789-2.251 | 0.523 | 0.212-1.292 | 0.154 | 0.021-1.106 | 1.046 | 0.602-1.818 | 2.156 | 1.098-4.236 | 0.222 | 0.054-0.905 |
| **Chronic brain ds** | 0.767 | 0.619-0.952 | 1.600 | 1.235-2.074 | 0.434 | 0.235-0.800 | 0.547 | 0.303-0.987 | 1.995 | 1.526-2.610 | 2.155 | 1.485-3.128 | 0.620 | 0.390-0.985 |
| **Malignancy** | 0.593 | 0.481-0.732 | 1.128 | 0.864-1.471 | 0.835 | 0.539-1.294 | 0.509 | 0.288-0.897 | 1.077 | 0.800-1.451 | 1.507 | 1.018-2.230 | 0.526 | 0.332-0.836 |
| **Autoimmune ds** | 0.724 | 0.373-1.405 | 1.697 | 0.798-3.608 | 0.898 | 0.276-2.926 | 0.437 | 0.060-3.194 | 2.204 | 1.076-4.517 | 2.040 | 0.716-5.815 | 2.779 | 1.262-6.122 |
| **Long-term steroid** | 1.247 | 0.401-3.876 | 1.086 | 0.237-4.971 | 1.805 | 0.405-8.039 | 0.001 | 0-Inf | 3.512 | 1.195-10.32 | 3.456 | 0.751-15.895 | 3.436 | 0.925-12.767 |
| **TNF blocker** | 0.483 | 0.097-2.395 | 1,983 | 0.399-9.846 | ─ | ─ | ─ | ─ | 2.605 | 0.524-12.94 | ─ | ─ | 1.725 | 0.212-14.06 |
| **AFB smear (+)** | 2.541 | 2.175-2.967 | 1.367 | 1.117-1.672 | 0.568 | 0.415-0.778 | 1.497 | 1.096-2.045 | 1.243 | 1.000-1.543 | 2.274 | 1.672-3.093 | 2.538 | 1.982-3.251 |
| **AFB culture (+)** | 1.888 | 1.628-2.190 | 1.359 | 1.107-1.669 | 0.648 | 0.506-0.830 | 1.038 | 0.767-1.404 | 1.112 | 0.906-1.363 | 1.694 | 1.202-2.386 | 1.941 | 1.465-2.569 |
| **Cavity (+)** | 1.647 | 1.386-1.957 | 0.747 | 0.580-0.961 | 0.792 | 0.567-1.107 | 2.812 | 2.059-3.840 | 0.797 | 0.608-1.043 | 1.215 | 0.849-1.738 | 2.621 | 2.026-3.392 |
| **Bilateral ds** | 1.371 | 1.185-1.587 | 1.725 | 1.420-2.095 | 0.716 | 0.544-0.942 | 1.230 | 0.906-1.671 | 1.419 | 1.156-1.742 | 2.029 | 1.493-2.748 | 2.156 | 1.685-2.759 |

Univariable analysis for development of each TB-related symptom was performed using logistic regression.

Abbreviation: ds, disease; AFB, acid-fast bacilli

Supplementary Figure S1. Venn diagram of symptoms related to tuberculosis


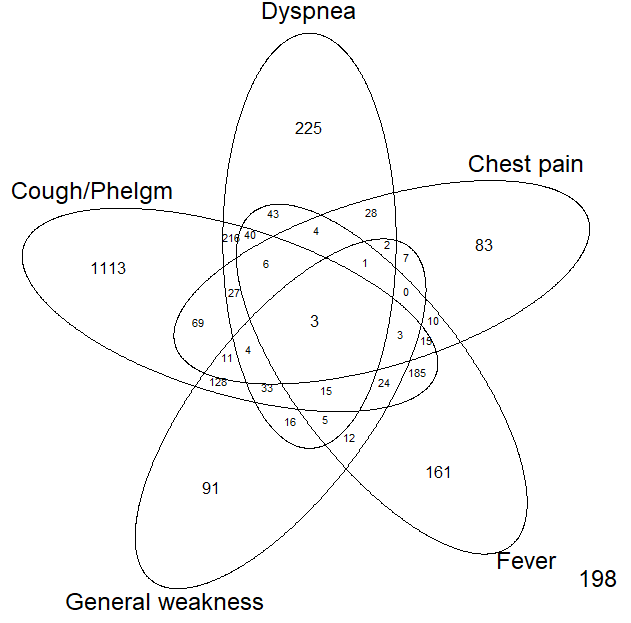


Only the top 5 symptoms are presented for Venn diagram

Supplementary Figure S2. Correlation matrix of between each symptom and demographic, microbiologic, and radiographic features


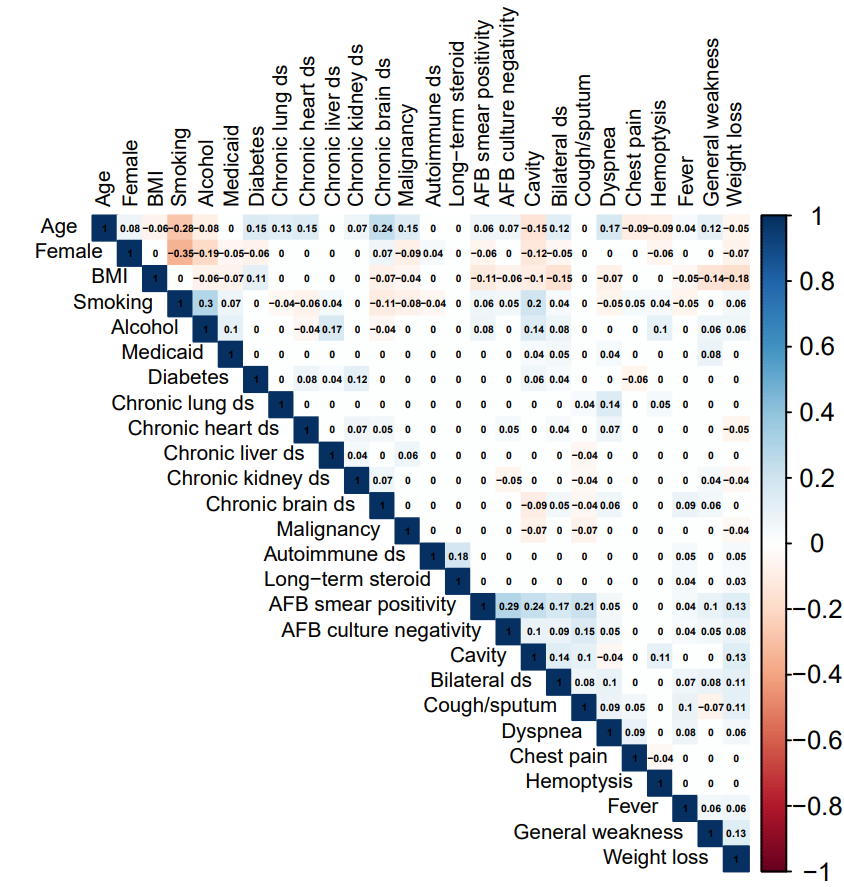


Correlation matrix was constructed using the Pearson correlation. Only significant correlations are demonstrated.

Supplementary Figure S3. Changes in the proportion of positivity of AFB (A) smear and (B) culture with increasing number of TB-related symptom presence


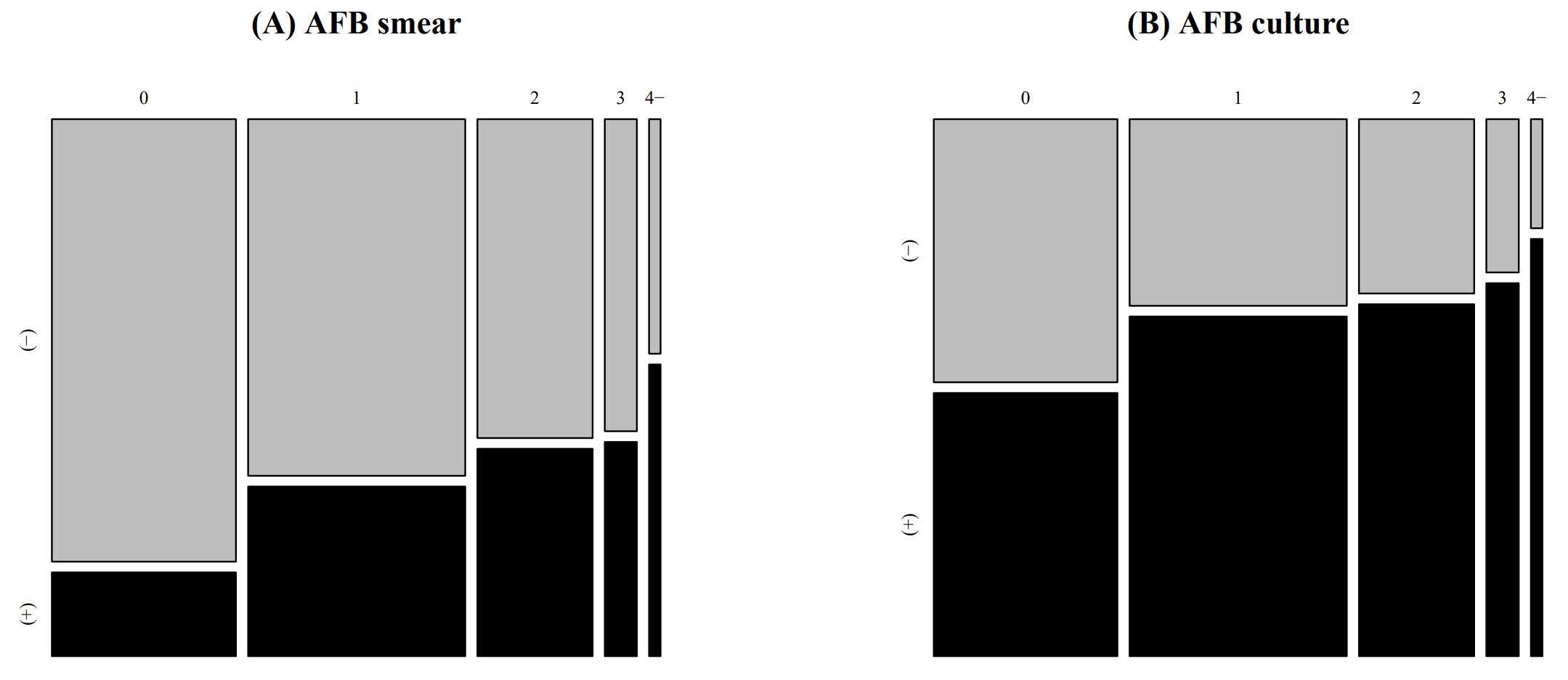

Supplement: Supplementary file 1 [file Data_Sheet_1.docx]
